# Oxidative Neurodegeneration Is Prevented by UCP0045037, an Allosteric Modulator for the Reduced Form of DJ-1, a Wild-Type of Familial Parkinson’s Disease-Linked PARK7

**DOI:** 10.3390/ijms10114789

**Published:** 2009-11-05

**Authors:** Koichiro Yamane, Yoshihisa Kitamura, Takashi Yanagida, Kazuyuki Takata, Daijiro Yanagisawa, Takashi Taniguchi, Takahiro Taira, Hiroyoshi Ariga

**Affiliations:** 1 Department of Neurobiology, Kyoto Pharmaceutical University, Kyoto 607-8414, Japan; E-Mails: ky04379@poppy.kyoto-phu.ac.jp (K.Y.); kaz@mb.kyoto-phu.ac.jp (K.T.); brain@mb.kyoto-phu.ac.jp (T.T.); 2 Department of Molecular Cell Biology, Interdisciplinary Graduate School of Medicine and Engineering, University of Yamanashi, Chuo 409-3898, Japan; E-Mail: taira@yamanashi.ac.jp (T.T.); 3 Department of Molecular Biology, Graduate School of Pharmaceutical Sciences, Hokkaido University, Sapporo 060-0812, Japan

**Keywords:** DJ-1, allosteric modulator, neuroprotection, anti-oxidative response, *in silico* virtual screening, *in vivo* rat brain, *in vitro* neuronal culture

## Abstract

Although a loss-of-function mutation has been identified in familial Parkinson’s disease PARK7, the wild-type of DJ-1 is known to act as an oxidative stress sensor in neuronal cells. Recently, we identified UCP0045037 as a compound that bound to the reduced form of DJ-1 by *in silico* virtual screening. In this study, we determined the neuroprotective effects of UCP0045037 against focal cerebral ischemia-induced neurodegeneration in rats. Hydrogen peroxide-induced cell death was significantly inhibited by UCP0045037 in both rat mesencephalic dopaminergic neurons and human normal SH-SY5Y cells. In contrast, DJ-1-knockdown SH-SY5Y cells lost the protective activity of UCP0045037. These results suggest that UCP0045037 interacts with endogenous DJ-1 and produces a neuroprotective response.

## 1. Introduction

DJ-1 was first discovered as a novel oncogene product in collaboration with activated small GTP-binding protein *ras* [1], and was later identified as a causative gene in the seventh type of familial Parkinson’s disease, PARK7 [2]. Wild-type DJ-1 has many functions, and can act as oxidative stress sensor, mitochondrial regulator, molecular chaperone, protease, regulator for gene transcription and mRNA stability, and also stimulatory factor for spermatogenesis and fertilization [3–6]. X-ray crystallographic and biologic analyses have shown that wild-type DJ-1 protein forms a homodimer. In addition, these analyses have also shown that a substitution of leucine at amino acid position 166 by proline (Leu166Pro-mutation) in DJ-1 protein, which was first found in a PARK7 patient, disrupts dimer formation, resulting in a loss of function [7–11]. Furthermore, the cysteine residues in wild-type DJ-1 protein are oxidized, and the isoelectric point (pI) of DJ-1 is shifted to being more acidic [12]. Thus, DJ-1 protein may play a key role in anti-oxidation and neuroprotection in neuronal cells [9,13–15].

**Figure 1.**
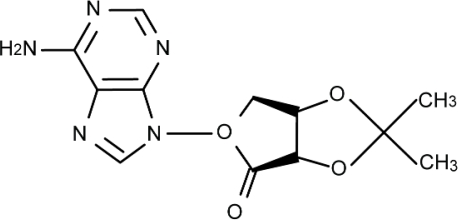
Chemical structure of UCP0045037.

Wild-type DJ-1 in human and rat has three cysteine (Cys) residues at amino acid numbers 46, 53 and 106 (Cys46, Cys53 and Cys106, respectively) [12]. A Cys residue is oxidized from the reduced form (-SH) to undergo sulphenation (-SOH), sulphination (-SO_2_H) and sulphonation (-SO_3_H), in order of oxidative development. Among these three Cys residues, Cys106 is the most sensitive to oxidative stress [12]. More recently, we used the X-ray crystal structure of DJ-1 at Cys106 with the reduced form and the three-dimensional coordinate data of about 30,000 chemical compounds in the University Compound Project (UCP) at the Foundation for Education of Science and Technology in Japan, and performed *in silico* virtual screening to search for DJ-1-binding compounds (DJ-1 ligands). Among the DJ-1 ligands identified *in silico*, UCP0045037 (Figure 1) had the highest binding constant (docking score) toward the pocket of the reduced Cys106 region and exhibited neuroprotective effects in a Parkinson’s disease model in rats, which were microinjected with 6-hydroxydopamine into the substantia nigra [16]. However, it is not yet known whether UCP0045037 is effective in brain stroke.

Cerebral ischemia occurs as a result of a local reduction or arrest of the blood supply, and leads to neuronal cell death in the ischemic region. Neurons and glial cells can be lethally damaged by several events, such as peri-infarct depolarization, which occurs within hours after the onset of ischemia, and the production of reactive oxygen species (ROS), which occurs immediately after ischemia/reperfusion, followed by more delayed post-ischemic inflammation and apoptosis, and these events are involved in the progression and expansion of brain injury [17,18]. This burst of ROS is involved in direct cytotoxic effects, including protein and lipid peroxidation, oxidative DNA damage and post-ischemic inflammatory injury through redox-mediated signaling pathways [19,20]. The oxidation of proteins can have wide-ranging damaging effects, such as disruption of the active sites of enzymes or alteration of the conformation of structural proteins. Thus, the components of ROS are thought to contribute to neuronal loss after ischemia/reperfusion [19,20]. Therefore, the control of ROS production is important for achieving neuroprotection against ischemia/reperfusion injury.

In the present study, to clarify the effects of UCP0045037 and DJ-1 on brain stroke and oxidative insult, we examined the effects of UCP0045037 on focal ischemia/reperfusion-induced oxidative brain damage in an *in vivo* neuronal model and on hydrogen peroxide (H_2_O_2_)-induced cell death in rat ventral mesencephalic neurons; or in normal and DJ-1-knockdown SH-SY5Y cells in an *in vitro* cultured neuronal model.

## 2. Results

### 2.1. Effect of DJ-1 Ligand in the in Vivo Rat Brain

**Figure 2.**
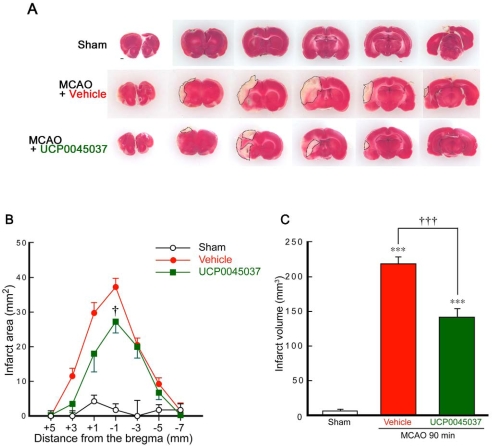
DJ-1 ligand UCP0045037 reduces infarct size after focal cerebral ischemia and reperfusion. (A) Representative photographs showing coronal brain sections at +5, +3, +1, −1, −3, −5, and −7 mm anterior-posterior from the bregma with TTC staining at 1 day after MCAO in sham-operated rats (n = 5) and MCAO-ischemic rats injected with sterilized physiological saline in the presence of the vehicle (4 μL, 1% DMSO, n = 5) or UCP0045037 (4 nmol/4 μL containing 1% DMSO, n = 5), at 30 min before MCAO (90 min). (B, C) Quantitative analysis of infarct area and volume, respectively. Data are the mean±SEM. Significance (Bonferroni/Dunn *post hoc* comparisons after ANOVA): ***P < 0.001 vs. sham-operated rats. †*P* < 0.05, †††*P* < 0.001 vs. vehicle-injected rats. Scale bar: 1 mm (Sham in A).

We previously found that neuronal death induced by occlusion of the middle cerebral artery (MCA) was significantly inhibited by the intrastriatal microinjection of recombinant protein of wild-type human DJ-1 [15]. Therefore, to clarify the effect of the DJ-1 ligand UCP0045037, which can bind to the pocket region at the reduced Cys106 of DJ-1 protein [16], in the *in vivo* ischemic brain, we performed an intrastriatal microinjection of this DJ-1 ligand and then subjected to the animals to 90 min of MCA occlusion (MCAO) and reperfusion (Figure 2).

Since UCP0045037 was hard to be solved by water, we prepared UCP0045037 solution at 1 mM (containing 1% dimethyl sulfoxide, DMSO) in sterilized physiological saline. At 24 h after MCAO, a marked decrease in the area and volume stained with 2,3,5-triphenyltetrazolium chloride (TTC) occurred in the ipsilateral cerebral cortex and striatum in rats, which were microinjected with 4 μL of sterilized physiological saline containing 1% DMSO (vehicle). In contrast, the area of TTC-staining lost was smaller with the intrastriatal microinjection of UCP0045037 (4 nmol/4 μL containing 1% DMSO) (Figure 2A). In the quantitative analysis, each infarct area was smaller and the total infarct volume was significantly reduced by the administration of UCP0045037, compared to that in vehicle-injected rats (Figures 2B and C).

### 2.2. Effect of DJ-1 Ligand in the in Vitro Neuronal Cultures

We have previously found that MCAO-induced focal ischemia retrogradely causes loss and damage of dopaminergic neurons in the rat substantia nigra, and results in behavioral and motor dysfunction [21]. It is well known that nigrostriatal dopaminergic neurons are specifically lost in the brains of patients with Parkinson’s disease. While, SH-SY5Y cells are frequently used as the human dopaminergic neuronal model [22]. In the present study, therefore, we focus effect of UCP0045037 on dopaminergic neurons, using *in vitro* rat and human dopaminergic neuronal models. In brief, we performed primary culture of dopaminergic neurons, which were prepared from the ventral two-thirds of the mesencephalon of rat embryos on the 16th days of gestation, as described previously [23]. We preliminarily obtained results that H_2_O_2_-induced cell death in normal SH-SY5Y cells was markedly inhibited by 10 μM UCP0045037, while moderately by 1 μM, but not by 0.1 μM (data not shown). Based on these observations, we examined effect of UCP0045037 at 10 μM on H_2_O_2_-induced cell death (and ROS production) in the rat primary-cultured neurons (and human SH-SY5Y cells) in Figure(s) 3 (and 4–6, respectively).

**Figure 3.**
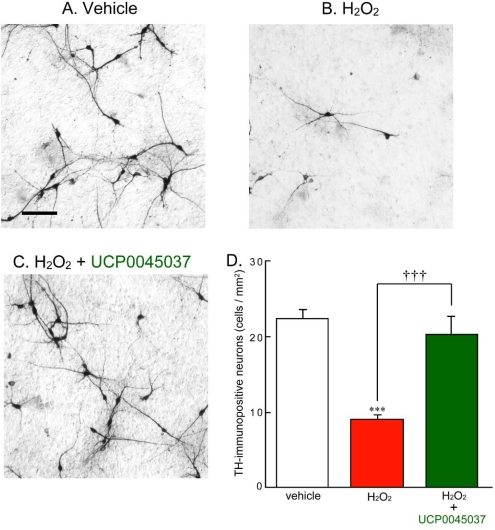
Protective effect of UCP0045037 on hydrogen peroxide-induced dopaminergic neuronal death. Rat ventral mesencephalic cultured neurons, prepared from day-16 embryos, were treated with 100 μM H_2_O_2_ in the presence of the vehicle (0.01% DMSO) or UCP0045037 at 10 μM (containing 0.01% DMSO) for 24 h. Subsequently, treated neurons were fixed and immunostained by anti-TH antibody and then the number of TH-immunopositive dopaminergic neurons was counted in the cultured area (1.9 mm^2^). (A, B, C) Immunoreactivity of dopaminergic neurons treated with vehicle, H_2_O_2_ alone, and H_2_O_2_ and UCP0045037, respectively. Scale bar: 100 μm. (D) Semi-quantitative analysis of dopaminergic neurons. Data are the mean±SEM of four determinations. Significance (Bonferroni/Dunn *post hoc* comparisons after ANOVA): ***p < 0.001 vs. vehicle-treatment. †††*p* < 0.001 vs. H_2_O_2_-treatment.

**Figure 4.**
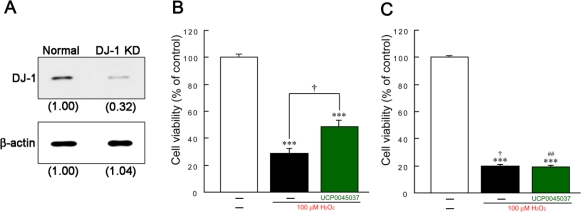
Effects of UCP0045037 on hydrogen peroxide-induced death of human SH-SY5Y cells. (A) Expression of endogenous DJ-1 protein. The intensity of the expression level in normal cells was set to 1.00. (B, C) Normal and DJ-1-knockdown SH-SY5Y cells (B and C, respectively) were treated with 100 μM H_2_O_2_ in the presence of the vehicle (0.01% DMSO) or UCP0045037 at 10 μM (containing 0.01% DMSO) for 24 h. Subsequently, cell viability was measured by a color metrical assay using 3–(4,5-dimethyl-2-thiazolyl)-2,5-diphenyltetrazolium bromide (MTT). Data are the mean±SEM of three determinations. Significance (Bonferroni/Dunn *post hoc* comparisons after ANOVA): ***p < 0.001 vs. vehicle-treatment. †*p* < 0.05 vs. H_2_O_2_-treatment in normal SH-SY5Y cells. ##*p* < 0.01 vs. H_2_O_2_-treatment in the presence of UCP0045037 in normal SH-SY5Y cells.

In primary culture of rat ventral mesencephalon, treatment with 100 μM H_2_O_2_ for 24 h significantly caused the death of dopaminergic neurons, which were immunostained by anti-tyrosine hydroxylase (TH) antibody (Figures 3A, B and D). Similarly, wild-type (normal) human SH-SY5Y cells also died upon H_2_O_2_-treatment for 24 h (Figure 4B). H_2_O_2_-induced death of either rat dopaminergic neurons or normal human SH-SY5Y cells was significantly inhibited by simultaneous treatment with 10 μM UCP0045037 (Figures 3C, D and 4B).

In DJ-1-knockdown SH-SY5Y cells, the expression of endogenous DJ-1 protein was suppressed by 68% (Figure 4A), and massive H_2_O_2_-induced cell death occurred, compared to the results in normal SH-SY5Y cells (Figure 4B and C). Interestingly, UCP0045037 did not protect against H_2_O_2_-induced death in DJ-1-knockdown cells (Figure 4C).

**Figure 5.**
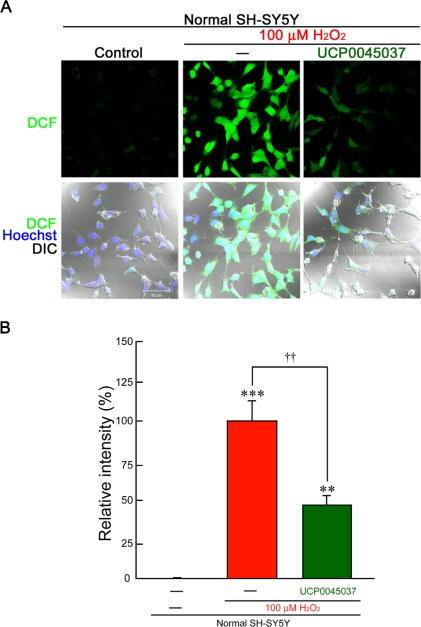
Effects of UCP0045037 on hydrogen peroxide-induced production of ROS in human SH-SY5Y cells. Normal SH-SY5Y cells were treated with 100 μM H_2_O_2_ in the presence of the vehicle (0.01% DMSO) or UCP0045037 at 10 μM (containing 0.01% DMSO) for 1 h. Subsequently, 5-(and-6)-chloromethyl-2′,7′-dichlorodihydro-fluorescein diacetate acetyl ester (CM-H_2_DCFDA) was added. Fluorescence of oxidized 2′,7′-dichlorofluorescein (DCF, green) was visualized, and then the intensity was measured as ROS production. (A) ROS production-dependent DCF fluorescence in normal SH-SY5Y cells. Nuclei were stained with Hoechst33258 (blue) and cellular images (cell shapes) were obtained by the difference interference contrast (DIC). (B) The relative fluorescence intensity was quantified by computerized image analysis. Data are the mean±SEM of five determinations, based on the intensity in normal cells treated with H_2_O_2_ alone as 100%. Significance (Bonferroni/Dunn *post hoc* comparisons after ANOVA): **p < 0.01, ***p < 0.001 vs. control. ††*p* < 0.01 vs. H_2_O_2_-treatment.

**Figure 6.**
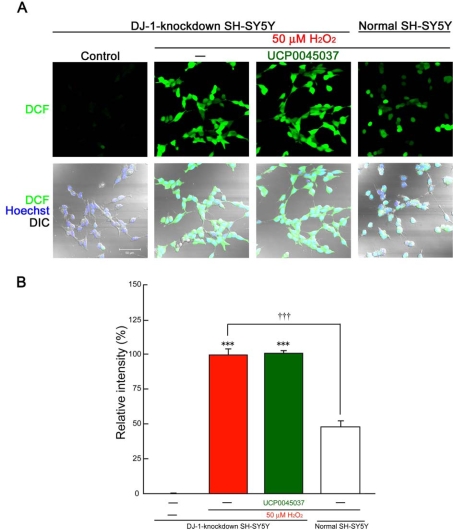
Loss of the inhibitory effect of UCP0045037 on hydrogen peroxide-induced production of ROS in DJ-1-knockdown cells. DJ-1-knockdown and normal SH-SY5Y cells were treated with 50 μM H_2_O_2_ in the presence of the vehicle (0.01% DMSO) or UCP0045037 at 10 μM (containing 0.01% DMSO) for 1 h, and then CM-H_2_DCFDA was added. Subsequently, ROS production-dependent DCF fluorescence was analyzed. (A) ROS production-dependent fluorescence in normal and DJ-1-knockdown SH-SY5Y cells, which were treated with 50 μM H_2_O_2_. Nuclei were stained with Hoechst33258 (blue) and cell shapes were obtained by DIC. (B) The relative fluorescence intensity was quantified by computerized image analysis. Data are the mean±SEM of three determinations, based on the intensity in DJ-1-knockdown SH-SY5Ycells treated with 50 μM H_2_O_2_ alone as 100%. Significance (Bonferroni/Dunn *post hoc* comparisons after ANOVA): ***p < 0.001 vs. control. †††*p* < 0.001 vs. H_2_O_2_-treatment in DJ-1-knockdown cells.

In normal SH-SY5Y cells, incubation with 100 μM H_2_O_2_ for 1 h induced marked production of intracellular ROS (Figures 5A and B), while the production was only slightly induced by 50 μM H_2_O_2_ (Figure 6A). In DJ-1-knockdown cells, even 50 μM H_2_O_2_ induced significant fluorescence intensity after 1 h (Figures 6A and B). On the other hand, treatment with 10 μM UCP0045037 significantly inhibited production of ROS, which had been induced by 100 μM H_2_O_2_, in normal SH-SY5Y cells (Figure 5B). Unfortunately, in DJ-1-knockdown cells, this DJ-1 ligand did not have an inhibitory effect on ROS production induced by 50 μM H_2_O_2_ (Figure 6B).

## 3. Discussion

Although oxygen is necessary for life, it paradoxically generates ROS, which are highly toxic to cells, as a by-product of its metabolism. In addition, ROS are massively produced in the brain after cerebral ischemia and reperfusion. Oxidative stress can be defined as an imbalance between ROS generation and the anti-oxidant capacity of a cell. Recent studies, together with our experiments, indicate that DJ-1 may sense oxidative stress. We previously found that treatment with recombinant human DJ-1 protein protected against cell death of nigrostriatal dopaminergic neurons in 6-hydroxydopamine-treated Parkinson’s disease model rats and MCAO-ischemic rats [9,15].

Protein oxidation indicates the trapping of oxygen atoms (Os) to amino acid residues, *i.e.*, methionine (Met) and Cys are oxidized to Met-SO (1O) and Cys-SO_3_H (3Os), respectively, and rat and human DJ-1 proteins have four Met and three Cys residues [24]. Therefore, if these amino acid residues are fully oxidized, one molecule of DJ-1 protein has a potential total of an additional 13Os. In addition, recent studies have indicated that a reduced form and/or partial oxidation at Cys106 induce DJ-1 activation, which may exert an antioxidative response, while massive oxidation may cause a loss-of-function of DJ-1 [13,24]. Thus, the reduced form (-SH) and/or moderate oxidation (-SOH and/or -SO_2_H) at Cys106 may produce active forms of DJ-1 (as a sensor of oxidative stress), while higher peroxidation (-SO_3_H) causes loss-of-function and produces the inactive form.

By *in silico* virtual screening, we recently identified UCP0045037 as a DJ-1-binding ligand, which can bind to the region around the reduced Cys106 form. In addition, direct binding between UCP0045037 and a recombinant wild-type DJ-1 protein (but not a mutated DJ-1 which has a substitution of Cys106 by serine, bovine serum albumin, or glutathione-*S*-transferase) was detected by a biosensor chip of a quartz crystal microbalance system [16], which suggests that the binding of UCP0045037 is relatively specific to the wild-type DJ-1 protein and its binding site may be the Cys106 region. Furthermore, matrix-assisted laser desorption/ionization (MALDI)-time of flight (TOF)/TOF-mass spectrometry (MS) analysis indicated that the H_2_O_2_-induced formation of hyperoxidative C106-SO_3_H was inhibited by UCP0045037 [16]. On the other hand, DJ-1 is relatively rich in neurons and glial cells, except for nigral dopaminergic neurons [14]. In addition to the presence of DJ-1 in the cytoplasm, mitochondria and nucleus, recent studies have reported that DJ-1 protein was detected in extracellular spaces, such as plasma and cerebrospinal fluids [25–28], suggesting that DJ-1 protein may function in both intracellular and extracellular spaces. Therefore, we consider that the amount of endogenous DJ-1protein is enough for the function of UCP0045037 in rat brain *in vivo* and in normal SH-SY5Y cells *in vitro*.

In the present study, we revealed that the intrastriatal microinjection of UCP0045037 inhibited neurodegeneration induced by 90 min of MCAO and reperfusion in rats. In rat mesencephalic primary culture, UCP0045037 protected against dopaminergic neuronal death induced by 100 μM H_2_O_2_. In normal SH-SY5Y cells, treatment with UCP0045037 reduced production of ROS and cell death induced by 100 μM H_2_O_2_. In DJ-1-knockdown cells, H_2_O_2_ at an even lower concentration (50 μM) significantly induced ROS production and cell death. However, DJ-1 knockdown caused a loss of the inhibitory effects of UCP0045037 toward ROS production and cell death. In DJ-1-knockdown SH-SY5Y cells in this study, endogenous DJ-1 protein partially remained (about 32%), but UCP0045037 did not affect in the knockdown cells. We presume that since residual 32% of DJ-1 protein might be almost completely SO_3_H-peroxidized by H_2_O_2_, simultaneous treatment with UCP0045037 could not inhibit SO_3_H-peroxidation of Cys106 in the knockdown cells. In normal SH-SY5Y cells, although DJ-1 protein was partially SO_3_H-peroxidized, the reduced and/or SOH/SO_2_H-oxidation forms of Cys106 might remain under treatment with UCP0045037. Therefore, UCP0045037 treatment may induce anti-oxidative and neuroprotective responses in normal cells. Based on these observations, we consider that endogenous DJ-1 protein is necessary for the antioxidant and neuroprotective effects induced by UCP0045037. In brief, UCP0045037 binds to DJ-1 protein as an allosteric modulator. Furthermore, since UCP0045037 binds to the reduced Cys106 region in wild-type DJ-1 protein, it is possible that UCP0045037 stabilizes the reduced DJ-1 form or mimics SOH/SO_2_H-oxidized Cys106, and inhibits peroxidation to inactive DJ-1 with SO_3_H-oxidized Cys106.

Recent studies have suggested that DJ-1 induces indirect antioxidant episodes, which are mediated through the stabilizing nuclear factor erythroid 2-related factor (Nrf2) by preventing association with the Kelch-like erythroid cell-derived protein with cap‘n’collar homology (ECH)-associated protein-1 (Keap1), and this activates antioxidant transcriptional responses, such as the induction and enhanced expression of NAD(P)H quinine oxidoreductase-1 (NQO1), heme oxigenase-1, superoxide dismutase-1, thioredoxin, and so on [29].

On the other hand, DJ-1 inhibits several apoptotic pathways, such as the pyrimidine tract-binding protein-associated splicing factor (PSF)/54-kDa nuclear RNA-binding protein (p54nrb) pathway [30] and/or apoptosis signal regulating kinase 1 (ASK1)/homeodomain-interacting protein kinase (HIPK1)/death-domain-associated protein (Daxx) pathway [31,32]. Thus, DJ-1 protein may induce: (i) an immediate antioxidant response by the direct trapping of oxygen atoms into amino acids; (ii) early indirect antioxidant responses, probably by the Nrf2-mediated induction of antioxidant enzymes/molecules, and (iii) delayed anti-apoptotic responses. Based on these observations, we consider that UCP0045037 may maintain and enhance DJ-1-induced anti-oxidative and anti-apoptotic activation and these responses may synergistically act to prevent both ROS production and neuronal cell death. Therefore, the interaction of 10 μM UCP0045037 with endogenous DJ-1 could inhibit 100 μM H_2_O_2_-induced ROS production and cell death in the *in vitro* culture model. These studies raise the possibility that UCP0045037 is a new type of neuroprotective drug for the treatment of brain ischemia.

## 4. Experimental Section

### 4.1. In Vivo Model of Rat Focal Cerebral Ischemia

Wistar rats were purchased from Japan SLC, Inc. (Hamamatsu, Japan). The animals were acclimated to and maintained at 23 °C under a 12-h light/dark cycle (lights on 08:00−20:00 hours). Rats were housed in standard laboratory cages and had free access to food and water throughout the study period. All animal experiments were carried out in accordance with the National Institutes of Health Guide for the Care and Use of Laboratory Animals, and the protocols were approved by the Committee for Animal Research at Kyoto Pharmaceutical University. Male Wistar rats weighing 260−300 g were used in the present study. Focal cerebral ischemia was induced by the intraluminal introduction of a nylon thread as described previously [15,33]. Briefly, animals were anesthetized with 4% halothane (Takeda Pharmaceutical, Osaka, Japan), and maintained on 1.5% halothane using a facemask. After a midline neck incision was made, 20 mm of 4-0 nylon thread with its tip rounded by heating and coated with silicone (Xantopren M; Heraeus Kulzer, Hanau, Germany) was inserted into the left internal carotid artery (ICA) as far as the proximal end using a globular stopper. The origin of the middle cerebral artery (MCA) was then occluded by a silicone-coated embolus. Anesthesia was discontinued, and the development of right hemiparesis with upper limb dominance was used as the criterion for ischemic insult. After 90 min of MCA occlusion (MCAO), the embolus was withdrawn to allow reperfusion of the ischemic region via the anterior and posterior communicating arteries. Body temperature was maintained at 37−37.5 °C with a heating pad and lamp during surgery. In the sham operation, a midline neck incision was made to expose the arteries, but the nylon thread was not inserted into the carotid artery.

### 4.2. Intrastriatal Drug Administration to Ischemic Rats

Male Wistar rats (SLC, Shizuoka), weighing approximately 300 g, were used. Under deep anesthesia (sodium pentobarbital, 50 mg/kg, i.p.), rats received a microinjection of UCP0045037 (4 nmol/4 μL) in the left striatum (coordinates: 1 mm anterior, 4 mm left lateral, and 5 mm ventral from the bregma). Sterilized physiological saline containing 1% dimethyl sulfoxide (DMSO) was used as the vehicle control in a final volume of 4 μL. After 30 min, left MCAO for 90 min and reperfusion were performed.

### 4.3. Measurement of Infarct Volume in Rat Ischemic Brain

At 24 h after MCAO, brains were removed and cut into 2-mm-thick coronal sections. These sections were immersed in 2% solution of 2,3,5-triphenyltetrazolium chloride (TTC; Wako Pure Chemical Industries, Osaka, Japan) in saline at 37 °C for 20 min, and then fixed in 4% paraformaldehyde in 100 mM phosphate buffer (PB) at 4 °C, and infarct areas and volumes were quantified.

### 4.4. Primary Neuronal Culture of Rat Ventral Mesencephalon

Cultures of the rat mesencephalon were established according to methods described previously [23]. The ventral two-thirds of the mesencephalon were dissected from rat embryos on the 16th days of gestation. The dissected regions included dopaminergic neurons from the substantia nigra and the ventral tegmental area but not noradrenergic neurons from the locus ceruleus. Neurons were dissected mechanically and plated out onto 0.1% polyethyleneimine-coated 24-well plates at a density of approximately 2,500,000 cells/well. The culture medium consisted of Dulbecco’s modified Eagle’s medium (DMEM) supplemented with 10% (v/v) fetal calf serum (FCS), 50 μg/mL penicillin and 100 μg/mL streptomycin and kept at 37 °C for two days in humidified 5% CO_2_/95% air, and DMEM containing 2% B-27 supplement (Invitrogen, Carlsbad, CA, USA) and 2 mg/mL aphidicolin (Sigma, St. Louis, MO, USA) without FCS from the third day onwards. To examine the effect of UCP0045037 on H_2_O_2_-induced neurotoxicity, the cultures were also incubated simultaneously with the medium with 2% B-27 supplement minus antioxidant (Invitrogen) containing 100 μM H_2_O_2_ in the presence of vehicle (0.01% DMSO) or 10 μM UCP0045037 (containing 0.01% DMSO) for 24 h on the 7th day of culture, and then fixed. Control experiments were sham operations, similar to the treatment but with the medium containing no drug. After fixation, cultured cells were incubated with anti-tyrosine hydroxylase (TH) antibody (diluted at 1:3,000; Chemicon International, Temecula, CA, USA) for approximately 24 h at 25 °C. The cultured cells were then incubated with avidin peroxidase (1:4,000; ABC Elite kit; Vector Laboratories, Burlingame, CA, USA) for 1 h at 25 °C. The cultured cells were washed several times with 100 mM phosphate-buffered saline containing 0.3% Triton X-100 (PBS-T) between each incubation, and labeling was revealed by 3,3′-diaminobenzidine with nickel enhancement, which yielded a dark blue color. TH-immunopositive neurons were then counted.

### 4.5. In Vitro Cell Culture and Establishment of DJ-1-Knockdown Cells

The human neuroblastoma cell line SH-SY5Y was cultured in DMEM supplemented with 10% FCS and kept at 37 °C in humidified 5% CO_2_/95% air. We established a DJ-1-knockdown SH-SY5Y cell line by a method that was essentially similar to that in mouse Flp-In3T3 cells [34]. In brief, the nucleotide sequence of the upper strand of the oligonucleotide used to construct an siRNA vector that targets the human DJ-1 gene is 5′-GGA TCC CGT CAA GGC TGG CAT CAG GAC AAT TGA TAT CCG TTG TCC TGA TGC CAG CCT TGA TTT TTT CCA AAA GCT T-3′. After oligonucleotides corresponding to the upper and lower strands of DNA were annealed, they were inserted into BamHI-HindIII sites of pRNA-U6.1/Neo. These plasmids were transfected into human SH-SY5Y cells by the calcium phosphate precipitation method, and the cells were cultured in RPMI-1640 medium in the presence of 400 μg/mL G418 for 14 days. Cells that were resistant to the drug were selected, and the intrinsic expression of DJ-1 was checked by western blotting with an anti-human DJ-1 antibody. In addition, normal and DJ-1-knockdown SH-SY5Y cells were treated with various concentrations of H_2_O_2_ (at 50 μM or 100 μM) in the presence of vehicle (0.01% DMSO) or 10 μM UCP0045037 (containing 0.01% DMSO) for 24 h.

### 4.6. Western Blotting Analysis

Cell lysates were diluted with Laemmli’s sample buffer and subjected to sodium dodecyl sulfate-polyacrylamide gel electrophoresis (15% polyacrylamide gels), and immunoblotting was then carried out using antibodies against human DJ-1 (1:5,000) and β-actin (10,000). For a semi-quantitative analysis, the bands of these proteins on radiographic films were scanned with a CCD color scanner (DuoScan; AGFA, Leverkusen, Germany), and then analyzed. Densitometric analysis was performed using the public domain program NIH Image 1.56 (written by Wayne Rasband at the US National Institutes of Health and available from the Internet by anonymous FTP from zippy.nimh. nih.gov.).

### 4.7. Assay of ROS Production

To detect H_2_O_2_-induced ROS production, we used a redox-sensitive dye, 5-(and-6)-chloromethyl-2′,7′-dichlorodihydro-fluorescein diacetate acetyl ester (CM-H_2_DCFDA; Molecular Probes) [22]. CM-H_2_DCFDA is readily taken up by cells. When it reacts with ROS, it is converted to oxidized 2′,7′-dichlorofluorescein (DCF), and subsequently illuminates at an excitation wavelength of 488 nm. After SH-SY5Y cells were prepared in uncoated glass-bottomed microwells (inner diameter, 18 mm), CM-H_2_DCFDA was added to the cell culture to a final concentration of 2 μmol/L for 10 min at 37 °C. After two rinses with serum-free medium, the fluorescence intensity of DCF was scanned under a confocal microscope (LSM410, Carl Zeiss, Jena, Germany). Since illumination at an excitation wavelength of 488 nm causes increased fluorescence due to oxidation of this dye [22], each field was exposed to light for exactly the same amount of time. Fluorescence intensity was quantified by computerized image analysis (WinRoof, Mitani. Fukui, Japan).

### 4.8. Measurement of Cell Viability

To evaluate cell viability, we performed a 3-(4,5-dimethyl-2-thiazolyl)-2,5-diphenyltetrazolium bromide (MTT; Dojindo Laboratories) assay as an index of surviving cells. In living cells, MTT is converted to formazan, which has a specific absorption maximum. SH-SY5Y cells, in which endogenous DJ-1 expression was normal or knocked-down by siRNA vector, were seeded at 30,000 cells/well in a 96-microwell plate, and treated with H_2_O_2_ 24 h after plating. At 24 h after treatment, the culture medium was changed to a medium containing 5 mg/mL MTT, and the cells were incubated for an additional 4 h. They were then mixed thoroughly with an equal volume of isopropanol/0.04 M HCl, and sonicated to completely dissolve formazan. Absorbance was then measured at 570 nm with a microplate reader (BioRad Laboratories, Hercules, CA, USA).

### 4.9. Statistical Analysis

Results are presented as the mean ± standard error of the mean (SEM). The significance of differences was determined by an analysis of variance (ANOVA). Further *post hoc* comparisons were performed using Bonferroni/Dunn tests (Stat View; Abacus Concepts, Berkeley, CA, USA).

## 5. Conclusions

This is the first report to show that UCP0045037 has a neuroprotective effect in an *in vivo* animal model of brain ischemia. UCP0045037 may bind to the reduced C106 region in endogenous DJ-1 protein and then maintain a DJ-1-induced neuroprotective response. The present results suggest that allosteric modulators for DJ-1 (DJ-1 ligands), such as UCP0045037, may be useful for neuroprotective treatment in cytoprotective therapy for various oxidative stress-mediated disorders.
